# COVID-19 Pandemic: An Opportunity for Universal Health Coverage

**DOI:** 10.3389/fpubh.2021.673542

**Published:** 2021-07-29

**Authors:** Chhabi Lal Ranabhat, Mihajlo Jakovljevic, Chun-Bae Kim, Padam Simkhada

**Affiliations:** ^1^Global Center for Research and Development, Kathmandu, Nepal; ^2^Manmohan Memorial Institute for Health Science, Department of Public Health, Kathmandu, Nepal; ^3^Institute of Comparative Economic Studies, Hosei University Chiyoda, Tokyo, Japan; ^4^Department of Global Health Economics and Policy, University of Kragujevac, Kragujevac, Serbia; ^5^Yonsei University, Wonju College of Medicine, Wonju, South Korea; ^6^Hongcheon County Hypertension and Diabetes Registration and Education Center, Hongcheon, South Korea; ^7^University of Huddersfield, School of Human and Health Sciences, Huddersfield, United Kingdom

**Keywords:** COVID-19, pandemic, universal health coverage, opportunities, challenge

## Introduction

Universal Health Coverage (UHC) is an ambitious goal in health service with sustainable development goal (SDG). Likewise this goal is a mean and end as well in comparison with other goals. The lessons that countries are currently learning from the COVID-19 pandemic all underscore that investing in health for all is not optional. Stable, equitable, prosperous and peaceful society and economy is only possible when no one is left behind. This global crisis created from COVID-19 pandemic could be an opportunity to seize the moment to make changes that benefit both UHC and health security.

### Foundations of Universal Health Coverage

#### Philosophical Foundation

The philosophical foundation of UHC is based with health care as fundamental human right and it is not limited with certain group or class of people (1). In the spirit of previous unifying concepts such as Health for All, basic health needs, and the Alma-Ata declaration, it presents a vision in which all citizens will enjoy a strong and efficient health system that spans preventive and curative medicine, affordable access to that health system, access to relevant medicines, and sufficient human resources. Current sustainable development goal (goal 3) universal health care has been modified and revised form of previous approach of primary health care, Millennium Development Goal (MDG) and other related movement in health care for justice. Previous developing countries health needs has been extended for all countries and accepted on the ground of justice and equity in SDG goal 3.

#### Sociopolitical Foundation

Different comparative research has shown that health coverage is associated with democratic political accountability (2). Access and quality of health care has been accepted as political agenda and every country political leaders gained power by this issue (2, 3). Likewise, health care is considered as social security those having poor financial contribution but higher need of health care like children, women, elder and poor economic status (4). The social development is possible where there is assurance of health care to all people.

#### Scientific Foundation

There are different health care practices locally, nationally and globally. UHC fundamentally owned modern health care i.e., allopathic health care, where there is proper diagnosis of illness, remedy based on research and evidence, principles of diseases control, eliminations and eradications (5). More likely, there are standard treatment and protocols to cure the diseases, adopted new interventions, drugs and technologies in health care. Successful policies and interventions are replicated with or without modifications to other communities and countries in terms of evidence based policies and practices. Beyond the allopathic approach, UHC included other alternative health care practice like Ayurveda, Naturopathy, Yoga etc. specially in health prevention and promotion (6). It assures that UHC could adopt alternative health care approach in future those have strong scientific ground.

#### Moral Foundation

Beyond the different foundations of UHC, another importation is moral or ethical. To achieve, UHC, only government effort is not sufficient for health and well-being of individual. So, individual health could be promoted by healthy behavior, preventive and promotive approach (7). Self-consciousness with own health, preventive measure on diseases and illness, promotive approach to restore health and contribution on government health financing by paying health insurance premium in time according to capacity to pay (8). In health care market, there are unethical practice in health care which are selection bias and moral hazard in both side; service provider and receiver and such things can be eliminated by high morale foundation of universal health care.

So, in any public health emergency and global pandemic like COVID 19, the collective and global effort is highly preferred. As the spirit of UHC, to exit from COVID 19 pandemic, it needs to address humanitarian way, balance socio-political context and promote scientific and moral ground intensively and parallel way.

## Impact of COVID-19

The first known human infections were in Wuhan, Hubei, China. A study of the first 41 cases of confirmed COVID-19, published in January 2020, reported the earliest date of onset of symptoms as 1 December 2019 (9, 10). Official publications from the WHO reported the earliest onset of symptoms as 8 December 2019 (11). Human-to-human transmission was confirmed by the WHO and Chinese authorities by 20 January 2020. At the middle of June 2021, total cases were about 18 million, death case were about 4 million, infection ratio is 23 thousand per million population, and death ratio was 500 per million population. By continent, North America about 20%, Europe 27%, Asia 30%, South America 17% and Africa 3% out of total cases (12). The new cases were continuously increasing and since February 2021, the new cases death are in decreasing trends.

Addressing to the COVID-19 pandemic has been and continues to be a huge challenge for many health systems and governments globally. In 2020 alone, there were an estimated 2.0 million lives lost to COVID-19, with some projections suggesting an additional 1.3 million lives could be lost in 2021 if mask wearing, social distancing, and hand washing are not strictly followed (13). Moreover, indirect health loss associated with lapses in essential health services such as vaccinations and access to safe emergency is substantial (14–16). To respond to the health crisis, governments around the world have implemented restrictions on travel and mass gatherings, required masks and quarantines, and rolled out and ramped up access to COVID-19 testing, case tracking, and, when possible, COVID-19 treatment (17). In addition, each government have fought to secure access to the first round of proven COVID-19 vaccines, with vaccination campaigns beginning in over 30 countries in 2020, including China, Israel, Russia, Mexico, the United States, and the United Kingdom (18).

It has been argued that health system actors should (i) embrace the notion of resilience as going beyond responses to sudden shocks, and encompassing everyday resilience, (ii) view health systems as comprised of both system software and hardware and (iii) conceptualize health system resilience as being about creative adaptation and transformation, not simply bouncing back (19). Following these perspectives, three levels of resilience can be applied to health systems: absorptive capacity, adaptive capacity and transformative capacity (20). In response to the current COVID-19 pandemic, we have seen that even previously high-performing health systems were becoming overstretched to adapt, casting a shadow on their ability to go further and transform (21, 22). This makes us wonder about the reliability of the metrics we have used so far to rate health systems, how can they be strengthened, and what other metrics that relate specifically to resilience need to be introduced. Being a COVID 19 as public health emergency and global pandemic (23), health care purchasing organizations are not able to add in their health insurance scheme and predict the health care cost by COVID 19. The job holders, who lost their job due to this pandemic automatically lost risk coverage (24). Due to this uncertainty, such pandemic addressing measures have been contingency approach than systematic.

## Possible Opportunities From Covid 19 Pandemic for Countries and World

Despite those human life and economic loss by COVID 19, this pandemic could be the great opportunity for the path to achieve universal health care in terms of the health related sustainable development goal. In other words, there is a great lesson learn from COVID 19 pandemic to cope with medical and public health emergency to individual, family, nation and global community. Furthermore, Coivd-19 pandemic has impacted on the mindset of policy makers and realized that there is need for more investment on health system. It has provided an opportunity to reassess priorities. This situation need to utilize as a strategic crisis to universal health care in the following areas:

### Reduce the Prevalence of Communicable Diseases and Promote Self-Care

COVID 19 is a disease by novel corona virus; a group of SARC virus and mostly effects on respiratory tract (25). To prevent its transmission, the applied preventive measures are use of mask, application of hand sanitizer and hand washing, maintain the physical and social distance and avoid to on crowding (26). This is an integrated approach and this approach significantly prevent from other communicable diarrheal and respiratory diseases. There is a high burden of communicable disease specially developing countries. Along with COVID 19, the incidence and prevalence of communicable diseases have been reduced and will be reduce in future too (23, 27). On the other hand such measures are cost effective and no need to consult the expertise. Individual and family member easily maintain this hygiene and prevent other diseases (28). Likewise, such hygiene promoting materials can be produced, transport and purchase easily in local situation too (29). Obviously, such approaches make individual more conscious to self-care and local health workers would be connected with people and easy access of health care.

### Promote Behavioral Modification for Non-communicable Diseases

Self-conscious and social distance approach makes behavioral change in everyday life and restrict physical distance (30). Gathering of many people, substance abuse and alcohol use, violence and gang fight etc. would be controlled. Those activities mostly happen in developed countries and large number of injuries and non-communicable disease would be reduced in log run. However, there are some debates to strict the social distance in terms of social functions and certain age group like elder population may particularly experience stress, increase physical dependency and emotional vulnerability (31).

### Health System Strengthening in Public Health Emergencies

COVID 19 pandemic is a great public health emergency in this century and national health system have been passive or they underestimated public health emergencies (32). After this pandemic, every countries health system have been alert and they stared to review their health policy, program, resources (33). Now, countries started to empower local health institutions and updated policy barriers and effective supply chain management of all health commodities. So, it is the proper time to strength the capacity of health system adopting global innovative approaches.

### Invention of Vaccine and Technologies

COVID pandemic is that situation where advance drugs and pharmaceuticals, surgery and sophisticated diagnostic approach could not effective. It demanded to invent vaccine and applicable for public in fast approach suspending some legal barriers. As a results, the vaccine against COVID 19 have been lunched within 9 months and in previous practice, there was at least 5 year to lunch such type of vaccines (34). Moreover, to detect the infection of corona virus, antibody test, plasma therapy etc. have been practiced. It provided great lesson to all scientist, policy makers and administrators to invent, trial and access the vaccines to people as far as possible.

### Strengthening Local to Global Partnership

Initially, there was accused for the transmission of COVID 19 but after some times, country's leader, policy makers, researchers and scientist have shared their progress through different partnership and collaborations (35). They have shared their progress through media, journals and press conference and global communities could have a hope and network in different setting.

### More Investment on Health Infrastructure and Human Resources for Health

Covid 19 pandemic has provided an opportunity to reassess priorities. Pandemic trend showed that public health emergency could be mitigated by well-infrastructure and sufficient, trained and committed human resources (36). Every government is now allocating more resources to health sectors.

## Challenges

### Mutation of Virus

Viruses constantly change through mutation and variations in the SARS-CoV-2 virus, due to evolution and adaptation processes, have been observed worldwide (37). While most emerging mutations will not have a significant impact on the spread of the virus, some mutations or combinations of mutations may provide the virus with a selective advantage, such as increased transmissibility or the ability to evade the host immune response. In UK, India and South Africa, new mutation of novel corona virus have been reported and such transmission is another great challenge. World Health Organization, indicated that Alpha, Beta, Gamma, Epsilon, Delta, Kappa and Eta variants has been reported and they have different transmissibility, virulence and antigenicity (38). This is a great challenge of this virus and existing loss due to COVID-19 impact.

### Balance the Demand and Supply of COVID 19 Vaccine

Almost 2.3 billion doses of COVID-19 vaccines could be rolled out worldwide through the COVAX Facility this year, according to a new supply forecast published this month (39). Of these, an expected 1.8 billion doses should be available to 92 lower-income economies. There is a large gap in demand and supply. It shows that there is big challenge to produce and supply of COVID-19 vaccine and among them low income countries those have no enough fund, poor supply chain system specially the cold chain for vaccine, internal governance and fragile health system would be highly affected.

### Access to Vaccine to the Poor People

The people in remote area, low socioeconomic status, poor access to health system, lower education, poor access with communication and media are the big challenges to get the coverage of COVAX. The elderly and disable people would be equally far from vaccines. Every government have this challenge should make a plan that how those groups ensure for vaccine coverage.

## Conclusion

There is continue loss of human life and wealth from COVID 19 and there is still threat and fear. The incidence and mortality is in increasing pace. In this situation it is necessary to continue preventive measures as far as possible because it is individual responsibility and cost effective approach. On the other hand there are some challenges for the access toward COVAX and need to provide them based on need other than economic, power and political influences. If so, this threat would be converted to opportunity in terms of improving health system strengthening, universal health care capacity build up on any public health emergency in coming future.

## Author Contributions

CR conceptualized, prepared, and updated the manuscript. MJ, C-BK, and PS reviewed and revised the paper. All authors contributed to the article and approved the final version.

## Conflict of Interest

The authors declare that the research was conducted in the absence of any commercial or financial relationships that could be construed as a potential conflict of interest.

## Publisher's Note

All claims expressed in this article are solely those of the authors and do not necessarily represent those of their affiliated organizations, or those of the publisher, the editors and the reviewers. Any product that may be evaluated in this article, or claim that may be made by its manufacturer, is not guaranteed or endorsed by the publisher.
